# Supplementary material for: The effectiveness of radial extracorporeal shock wave therapy vs transcutaneous electrical nerve stimulation in the management of upper limb spasticity in chronic-post stroke hemiplegia–A randomized controlled trial
Source: PLoS One. 2023 May 26;18(5):e0283321. doi: 10.1371/journal.pone.0283321 (PMC10218748; doi:10.1371/journal.pone.0283321)
Supplement: S1 Fig — (DOC) [file pone.0283321.s003.doc]

**
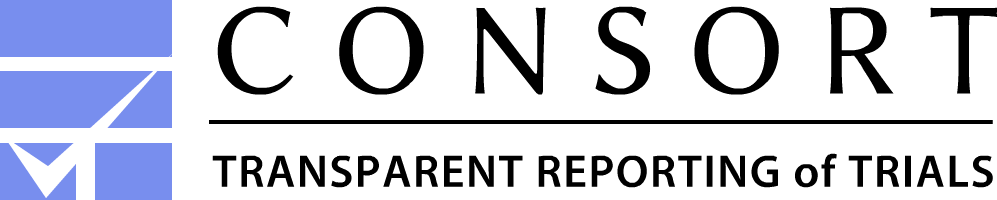
**

**CONSORT 2010 Flow Diagram**

**Allocation**

**Analysis**

**Follow-Up**

**Enrollment**

Assessed for eligibility (n=460)

Excluded (n=273)

♦  Not meeting inclusion criteria (n=268)

♦  Declined to participate (n=0)

♦  Other reasons (n=05)

Analysed (n=53)
♦ Excluded from analysis (give reasons) (n=0)

Lost to follow-up (give reasons) (n=0)

Discontinued intervention (give reasons) (n=0)

Allocated to intervention rESWT (n=54)

♦ Received allocated intervention (n=53)

♦ Did not receive allocated intervention (Failed to participate in four-week treatment protocol) (n=1)

Lost to follow-up (give reasons) (n=0)

Discontinued intervention (give reasons) (n=0)

Allocated to intervention TENS (n=54)

♦ Received allocated intervention (n=53)

♦ Did not receive allocated intervention (Failed to participate in four-week treatment protocol) (n=1)

Analysed (n=53)
♦ Excluded from analysis (give reasons) (n=0)

Randomized (n=187)
