# Supplementary material for: The effectiveness of radial extracorporeal shock wave therapy vs transcutaneous electrical nerve stimulation in the management of upper limb spasticity in chronic-post stroke hemiplegia–A randomized controlled trial
Source: PLoS One. 2023 May 26;18(5):e0283321. doi: 10.1371/journal.pone.0283321 (PMC10218748; doi:10.1371/journal.pone.0283321)
Supplement: S1 File — (DOCX) [file pone.0283321.s004.docx]

## Study Protocol

## Research Design

This was a prospective, randomized, comparative, single-blind study between radial extracorporeal shock wave therapy (rESWT) and transcutaneous electrical nerve stimulation (TENS) for treating upper limb spasticity of chronic post-stroke hemiplegia. There were no adverse effects that arose from both the rESWT and TENS.

## Study Site

Study subjects were enrolled under the supervision of Prof. L. P. M. M. K. Pathirage, Consultant Physician, Teaching Hospital, Peradeniya (Professor in Medicine, Department of Medicine, University of Peradeniya). 'Peradeniya stroke registry' was the data source to identify and recruit patients who were diagnosed as chronic stroke patients (first episode) with spasticity. Patients were asked to be present and the study took place at the Patients' Service Unit, Department of Physiotherapy, Faculty of Allied Health Sciences, University of Peradeniya due to the availability of the rESWT and TENS machines.

## Inclusion Ccriteria and Exclusion Criteria

### *Inclusion Criteria*

- Subjects diagnosed as stroke patients by physician
- Ischemic or hemorrhagic type of stroke
- Both male and female patients aged between 40 to 70 years
- Patients with the first-ever stroke for more than six months resulting in hemiplegia
- Able and willing to participate in four weeks study and to sign the consent form

### *Exclusion Criteria*

- Thrombosis type of stroke patients
- With reflex sympathetic dystrophy/ severe shoulder subluxation/ Shoulder dislocation/ Contracture in the affected UL/ Fixed deformity of hand/ Shoulder hand syndrome
- Patients who have received botox injection or acupuncture within the past 6 months to the affected UL
- Complete sensory loss of the upper limb
- Unstable medical condition like severe hypertension, convulsion
- Visually impaired patients
- Behavior problems

## Sampling Method

To reduce a type II error and increase the power, a preliminary power analysis using G∗power 3.1.9.2 computer program, based on t-test, means: Wilcoxon-Mann-Whitney test (two groups); power (1 − β) = 0.85; α = 0.05; effect size = 0.5 were used to calculate the sample size. It indicated that a total sample of 108 people would be needed^1^.

## Variables

- Stroke type
- Gender
- Affected side
- Affects the dominant side

## Data Collection and Data Collection Tools

Ethical clearance (2019/EC/35) for this study was obtained from Ethics Review Committee, Faculty of Medicine, University of Peradeniya (Annexure 01), and it was registered under Sri Lanka Clinical trial Registry with the registration number of SLCTR/2019/040 (Annexure 02) to conduct as a clinical trial. Patients were recruited based on 'Peradeniya Stroke Registry (PSR)'; 544 patients registered from January 2017 to the end of June 2019, which were considered for this study. As the first step, 54 patients who were given thrombolysis, 22 deaths, and eight (8) patients who were diagnosed with both stroke types or non-mentioned diagnosis were excluded from the PSR. Remained total number of 460 were contacted by the principal investigator to sort out the eligibility (inclusion and exclusion criteria) and their consent for this study. Among them, five deaths were recorded and 268 had better recovery. Among filtered 187 patients, 108 patients, both genders, the age range of 40-70 years, were randomly selected for this study. There were 76 ischemic stroke and 32 hemorrhagic stroke patients. Two ischemic stroke patients were unable to complete the whole treatment protocol, therefore they were excluded from the study. Finally, the sample size was 106 chronic stroke patients. They were divided into two main groups' Ischemic stroke' and 'Hemorrhagic stroke'. They were further divided into four groups as follows.

G11 = Ischemic stroke/ Experimental group

G12 = Ischemic stroke/ Control group

G21 = Hemorrhagic stroke/ Experimental group

G22 = Hemorrhagic stroke/ Control group

The randomization process for the ischemic group or hemorrhagic group was followed separately. The first ischemic patient and the first hemorrhagic patient were selected to either experimental or control group by 'Envelop method' (same-sized and same-colored two envelops)^2^. There were two pieces of paper, one was written as '1' and the other as '2'. Each envelope contained only one piece of paper. The patient selected either '1' or '2', but he/ she didn't know what is meant by '1' or '2'. Only the principal investigator knows that '1' is indicated for 'Experimental group' and '2' is indicated for the 'Control group'.

1 = Experimental group (treatment with rESWT)

2 = Control group (treatment with TENS)

X = Experimental group

Y = Control group

j implies j^th^ treatment ; j = 1,2

X_ij_ implies i^th^ patient with j^th^ treatment

Y_ij_ implies i^th^ patient with j^th^ treatment

If the first ischemic stroke patient was selected to the experimental group (he/ she was denoted as X_11_), the second ischemic stroke patient was included in the control group (he/ she was denoted as X_22_). 3^rd^, 4^th^, and remaining patients (76 of total patients) were denoted as follows.

X_31,_ X_42,_ X_51,_ X_62,_ X_71,_ X_82,_ X_91, …………………….._ X_762_

Therefore;

G11 = X_11,_ X_31,_ X_51,_ X_71,_ X_91, …………………….._ X_751_

G12 = X_22,_ X_42,_ X_62,_ X_82,_ X_102, …………………….._ X_762_

If the patients were selected from the hemorrhagic stroke group, the same procedure above was followed to allocate to the experimental or control group (total 32 patients).

G21 = Y_11,_ Y_31,_ Y_51,_ Y_71,_ Y_91, …………………….._ Y_311_

G22 = Y_22,_ Y_42,_ Y_62,_ Y_82,_ Y_102, …………………….._ Y_322_

Data were collected by the principal investigator for one year and three months through a neurological assessment and a clinical examination of the consented subject. Before being assigned to one of two treatment groups, all subjects received a description of the study. After the participants have signed the informed written consent they were randomly assigned to the rESWT treatment group or the TENS treatment group. The experimental group was treated with rESWT and the control group was treated with TENS. All the subjects had gone through examination criteria to assess the spasticity level and the functional ability level of their affected upper limb. The modified Ashworth scale was used to assess the spasticity grading. Fugl-Meyer motor assessment scale for upper limb and action research arm test were used to assess the functional ability of the affected upper limb. Collected data from the subjects are kept confidential and accessible only to the researcher and the supervisors. Subjects were not exposed unnecessarily throughout the clinical examination.

**Outcome measures:**

- Modified Ashwath scale (MAS)
- Voluntary control grading (VCG)
- Fugl - Meyer motor assessment of physical performances – upper limb (FMA-UL)
- Action research arm test (ARAT)

**Measurements:**

***Spasticity grading: Modified Ashworth Scale (MAS):*** The MAS is widely and consistently used in clinical practice and research to evaluate spasticity. MAS has good validity in patients with chronic stroke^3^. The scale is graded in 6 stages (0, no increase in tone; 1, slightly increased tone, giving a catch/release or minimal resistance at the end ROM; 1^+^, slightly increased tone, giving a catch followed by minimal resistance throughout the remainder (less than half) of the ROM; 2, more markedly increased tone through most of the ROM but the affected part easily moved; 3, considerably increased tone and passive movement difficult; and 4, limb rigid in flexion or extension). For the convenience of statistical analysis, MAS grade 1^+^ was point 2; grades 2, 3, and 4 were respectively matched to 3, 4, and 5. (Figure 1)^4,5^


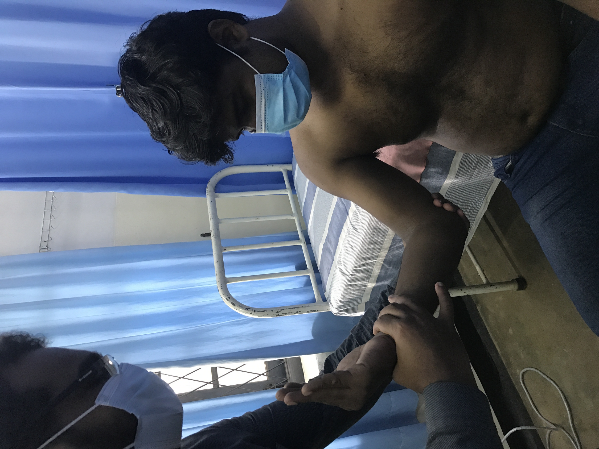


Figure 01: Assessing the spasticity of upper limb

***Voluntary control grading of the upper limb:*** This grading system is for assessing the synergic patterns of voluntary movements. This scale has seven grades from 0 to 6. (0, no contraction; 1, a flicker of contraction present or the initiation of movement; 2, half ROM is synergy or abnormal pattern; 3, full ROM is synergy or abnormal pattern; 4, initial half range is performed in isolation; 5, full ROM in isolation but goes into the pattern when resistance is offered and the latter half in pattern; 6, full range of motion isolation against resistance). (Figure 2)^6^


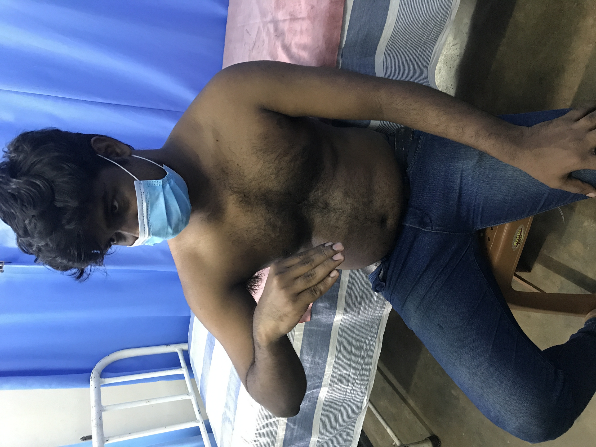


Figure 02: Assessing the VCG of upper limb

***Hand function: Fugl-Meyer Assessment (FMA):*** The FMA assesses motor function recovery after stroke and consists of 33 and 17 performance items in the upper and lower limbs, respectively. The scores ranging from 0–2 (0-unable to perform, 1-partial ability to perform, and 2-near-normal ability to perform), therefore, there are 66 total marks for the upper limb component^7^. The items that measure wrist control and hand function have been revealed to have excellent intrarater reliability and high interrater reliability^8^.

***Hand function: Action research arm test:*** The Action Research Arm Test (ARAT) is an evaluative measure to assess specific changes in limb function among individuals who sustained cortical damage resulting in hemiplegia^9^. It consisted of four components (grasp-6, grip-4, pinch-6, gross motor-4) which assesses a patient's ability to handle objects differing in size, weight, and shape and therefore can be considered to be an arm-specific measure of activity limitation. The scores ranging from 0-3 (0-can perform no part of test, 1-performs test partially, 2-completes test but takes abnormally long or has great difficulty, and 3-performs test normally), therefore, there are 57 total marks for the upper limb component^10-13^.

**Procedure:**

Initially, all the grading of the spasticity level, voluntary motor control, hand functions, and other measurements and data were gained through the assessment and clinical examination. If the consented subject belongs to the control group, they received TENS, frequency of 100 Hz over Teres major, Brachialis, Flexor carpi ulnaris, and Flexor digitorum profundus muscles^14^, 15 minutes for each. If the consented subject belongs to the experimental group, they received rESWT to the middle of the muscle belly, 1500 shots per muscle, frequency of 5Hz, energy of 0.030 mJ/mm, over Teres major, Brachialis, Flexor carpi ulnaris, and Flexor digitorum profundus muscles. All the patients received TENS or rESWT once a week for four weeks continuously according to the group to which he or she was assigned. All the subjects were assessed for their voluntary movement grading for the upper limb, spasticity grading for upper limb and their hand function grading at baseline (before the first treatment-T0), immediately after the first TENS or rESWT treatment has been given (T1), and at the end of the four weeks treatment session (T2).

***Application of TENS:***

The patient was in a seated position with the affected arm may or/ may not rest on a pillow. TENS was applied over mentioned muscles in 100 Hz frequency for 15 minutes. The passive electrode was placed at the cervical region (C7) and the active electrode was placed on the middle of the muscle belly. (Figure 3-6)


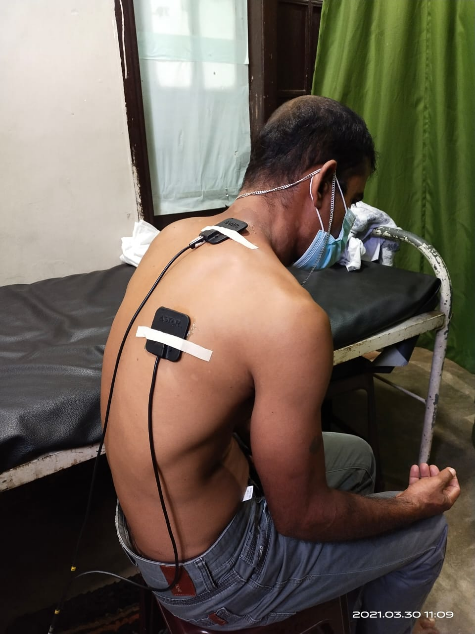


Figure 03: TENS application over Teres major muscle


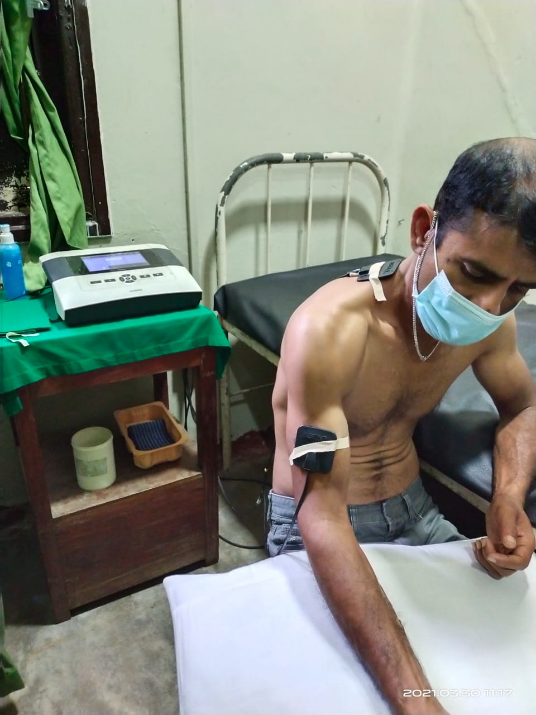


Figure 04: TENS application over Brachialis muscle


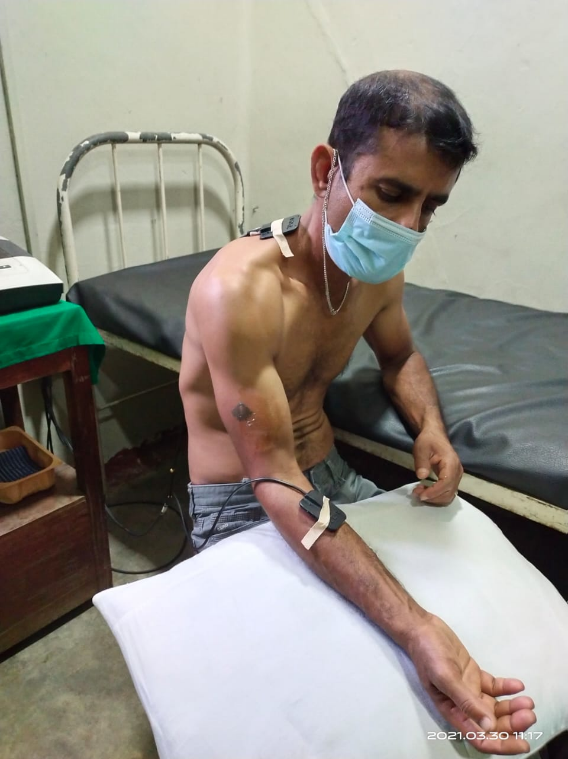


Figure 05: TENS application over Flexor carpi ulnaris muscle


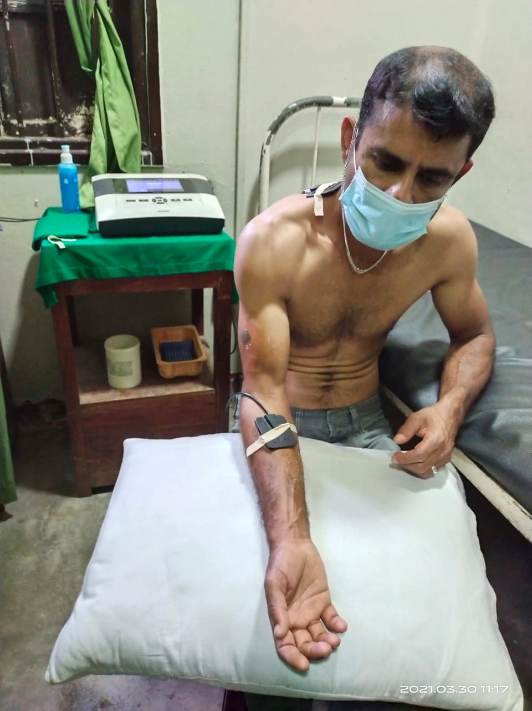


Figure 06: TENS application over Flexor digitorum profundus muscle

***Application of rESWT:***

The patient was in a seated position with the affected arm may or/ may not rest on a pillow. Shock wave therapy (rESWT) was applied to the middle of the muscle belly, 1500 shots per muscle, frequency of 5Hz, energy of 0.030 mJ/mm, over the above-mentioned muscles. The handle/ wand of the therapy unit was perpendicular to the skin. (Figure 7-10)


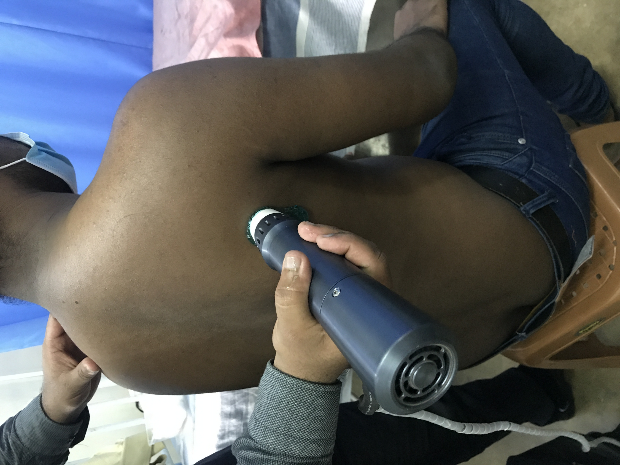


Figure 07: rESWT application over Teres major muscle


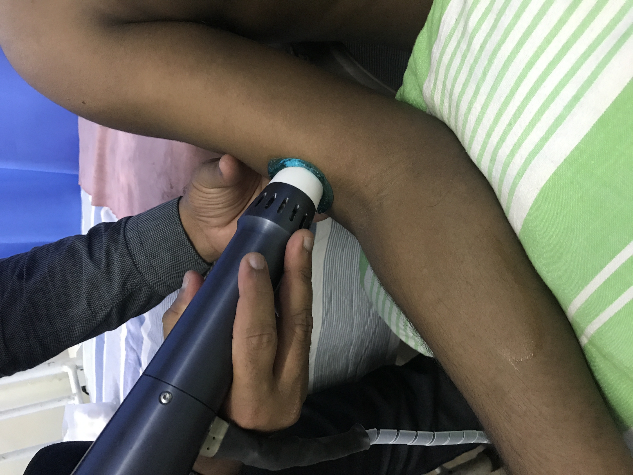


Figure 08: rESWT application over Brachialis muscle


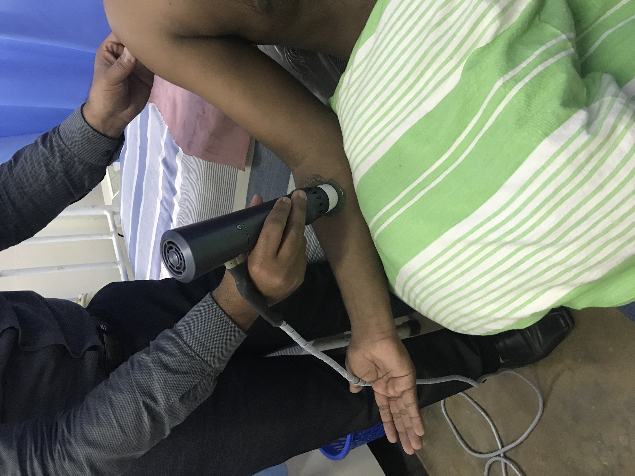


Figure 09: rESWT application over Flexor carpi ulnaris


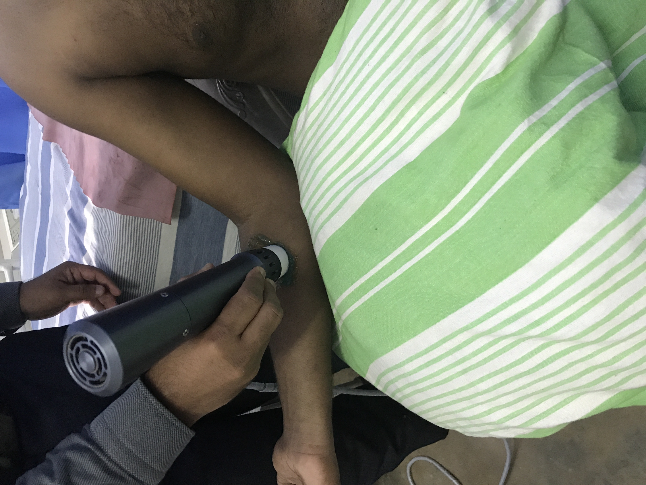


Figure 10: rESWT application over Flexor digitorum profundus

## 2.7 Data Entry and Statistical Analysis

All the collected data were fed into a Microsoft Excel sheet and statistical analysis was conducted using SPSS 22. Median values were calculated for spasticity grading and voluntary control grading as they are categorical variables. Then, a non-parametric test, Wilcoxon signed rank test (*z*-value and *p*-value), was used to identify whether there are any statistically significant median difference in pairs of time points (T0 & T1, T0 & T2, and T1 & T2). Paired sample t-test (*p*-value) was used to analyze the statistical significance of mean values of two continuous variables; grading for Fugl-Mayer assessment for upper limb and action research arm test. The mean/ median differences in the outcome variables were calculated for the experiment and control groups' pre and post-treatment. Comparisons of the outcome variables were conducted among T0 with T1 and T2, and T1 and T2. Along with paired sample t-test, paired sample correlations were tested which showed the bivariate Pearson correlation coefficient (with a two-tailed test of significance) for each pair of variables entered (*r-*value and *p*-value).  A probability level of 0.05 was selected as the criterion for significance in Wilcoxon signed ranks test and paired sample t-test, and 0.001 was selected in paired sample correlation test.

*Wilcoxon signed rank test*

*p*< 0.05 – population has a median distinct from the hypothetical value entered (there is a statistically significant difference between median values of two time points)

*p*> 0.05 – population median is not distinct from the hypothetical median (there is no statistically significant median difference between two time points)

*Paired sample t-test*

*p*< 0.05 – there will be a statistically significant difference between the mean values of two time points

*p*> 0.05 – there will be no statistically significant difference between the mean values of two time points

*Paired sample correlation*

The null hypothesis (*H*_0_) and alternative hypothesis (*H*_1_) of the significance test for correlation can be expressed in the following ways in two-tailed significance test.

*H*_0_: *ρ* = 0 ("the population correlation coefficient is 0; there is no association")
*H*_1_: ρ ≠ 0 ("the population correlation coefficient is not 0; a nonzero correlation could exist")

And;

*p*< 0.001 – there will be a significant linear relationship/ correlation between the two pairs (statistically significant)

*p>* 0.001 – there will be no significant linear relationship/ correlation between two pairs (statistically significant)

-The direction of the correlation:

- -1 : perfectly negative linear relationship
- 0 : no relationship
- +1 : perfectly positive linear relationship

-The strength of the correlation:

- 0.1 < | *r*| < 0.3 … small / weak correlation
- 0.3 < | *r*| < 0.5 … medium / moderate correlation
- 0.5 < | r | ….…… large / strong correlation

As spasticity (Modified Ashwath scale-MAS) and voluntary control of the affected upper limb (Voluntary control grading scale-VCG) were categorical variables; the reduction of spasticity and progress of the voluntary control were measured as how many grades were improved.

As the functional capacity of the affected upper limb was a continues variable (Fugyl-Mayer Assessment of upper limb-FMA-UP and Action Research Arm Teat-ARAT); the improvement of the functional capacity was measured as percentages compared.

*Fugyl-Mayer assessment for upper limb (FMA-UL)*

Improved mean difference as a percentage

= post-mean score at time point_b_ (-) pre-mean score at time point_a_ X 100

66

*Action research arm test (ARAT)*

Improved mean difference as a percentage

= post-mean score at time point_b_ (-) pre-mean score at time point_a_ X 100

57

Following comparisons were conducted according to the treatment option, stroke type, gender, affected side, and affecting dominant side in included subjects. All the treatment groups were analyzed separately for the assessments taken at T0, T1, and T2. Pairwise comparisons were conducted between To & T1, T0 & T2, and T1 & T2. To analyze the improvements of the grades and the scores from baseline to other time points. **References**

1. Faul F, Erdfelder E, Lang AG. et al. G^∗^Power 3: A flexible statistical power analysis program for the social, behavioral, and biomedical sciences. Behav Res Methods. 2007;39:175–191.
2. Torgerson DJ, Roberts C. Randomization methods: concealment. BMJ*.* 1999;319(7206): 375-376.
3. Lin FM & Sabbahi M. Correlation of spasticity with hyperactive stretch reflexes and motor dysfunction in hemiplegia. Arch Phys Med Rehabil*.* 1999;80:526–530.
4. Santamato A, Notarnicola A, Panza F. et al. SBOTE study: extracorporeal shock wave therapy versus electrical stimulation after botulinum toxin type A injection for post-stroke spasticity-a prospective randomized trial. Ultrasound Med Biol.  2013;39:283–291.
5. Kim YW, Shin JC, Yoon JG. et al. Usefulness of radial extracorporeal shock wave therapy for the spasticity of the subscapularis in patients with stroke: a pilot study. Chin Med J (Engl). 2013;126:4638–4643.
6. Das P. Voluntary motor control. [Cited 30 June 2017]. Available from: <http://www.physiotherapy-treatment.com/voluntary-motor-control.html>
7. Fugl-Meyer AR, Jaasko L, Leyman I. et. al. The post-stroke hemiplegic patient. 1. A method for evaluation of physical performance. Scand J Rehabil Med*.*1975;7:13–31.
8. Duncan PW, Propst M & Nelson SG. Reliability of the Fugl-Meyer assessment of sensorimotor recovery following cerebrovascular accident. Phys Ther. 1983;63:1606–1610.
9. Lyle RC. A performance test for assessment of upper limb function in physical rehabilitation treatment and research. Int J Rehabil Res. 1981;4:483-492.
10. Carroll D. A quantitative test of upper extremity function. J Chronic Diseases. 1965;18:479-491.
11. De Weerdt WJG & Harrison MA. Measuring recovery of arm-hand function in stroke patients: a comparison of the Brunnstrom-Fugl-Meyer test and the Action Research Arm Test. Physiotherapy Canada. 1985;37:65-70.
12. Crow JL, Lincoln NNB, Nouri FM & De Weerdt W. The effectiveness of EMG biofeedback in the treatment of arm function after stroke. International Disability Studies. 1989;11:155-160.
13. Platz T, Pinkowski C, Wijck VF, Kim IH, Bella DP & Johnson G. Reliability and validity of arm function assessment with standard guidelines for the Fugl-Mayer test, action research arm test and box and, block test: a multicenter study. Clin Rehabil. 2005;19(4):404-411.
14. Dauzvardis MF, McAutly JA, Espiritu B, Lee D, Sadowski S, Klitz G. et. al. [Cited 6 July 2017]. Master muscle list. Loyola university medical education network. Available from: http://www.lumen.luc.edu/lumen/meded/grossanatomy/dissector/mml/mmlregn.htm
