# Supplementary material for: The effectiveness of radial extracorporeal shock wave therapy vs transcutaneous electrical nerve stimulation in the management of upper limb spasticity in chronic-post stroke hemiplegia–A randomized controlled trial
Source: PLoS One. 2023 May 26;18(5):e0283321. doi: 10.1371/journal.pone.0283321 (PMC10218748; doi:10.1371/journal.pone.0283321)
Supplement: Attachment — Submitted filename: Response to reviewers comments.docx [file pone.0283321.s005.docx]

MKI Dilhari Senarath

Dept. of Physiotherapy

Faculty of Allied Health Sciences

University of Peradeniya

Peradeniya, 20400, Sri Lanka

10/06/2022

Dr. Emily Chenette

Editor-in-Chief

The Journal of PLOS ONE

Dear Dr. Chenette,

**RE:** **Response to reviewers’ comments for the article titled ‘The effectiveness of radial Extracorporeal Shock Wave Therapy vs Transcutaneous Electrical Nerve Stimulation in the management of Upper Limb spasticity in chronic-post stroke hemiplegia – A randomized controlled trial’**

Here I am attaching the table regarding the authors’ responses to reviewers’ comments one by one on the above-mentioned article.

|  | Reviewers’ comment | Authors response | Page number |
| --- | --- | --- | --- |
| 1 | The authors should improve the English grammatical | Thank you for the comment. After addressing all the other comments, we corrected the grammatical errors by a language expert. | Throughout the manuscript |
| 2 | The authors should describe the method of **randomization**, do they use computerized block randomization? How do they conceal the treatment allocation? | The randomization process was done in different steps. It is further described under ‘Methodology-Randomization’. We added more details to the randomization process and treatment allocation. | Page 6 |
| 3 | The authors should add a **baseline characteristic table**. It is very important to make sure the baseline characteristics similar | A baseline characteristic table is added under the Results section. | Page 9  Table 1 |
| 4 | It is better to add the **time horizon from stroke onset** to subjects’ recruitment | We described patient recruitment under a new topic as ‘Methodology-Study Design and Patient Recruitment’.  It describes the time period of patient allocation and stroke onset. | Page 4 |
| 5 | It is better to **concise in introduction and give more detail in method** | Reduce the content of the introduction and add more details in the Methods section.  We added study design and patient recruitment under the Methodology section and further described the randomization process. | Page 3, 4, 6 |
| 6 | Please add the **limitation of your study** | A new paragraph of ‘Limitations’ is included in the manuscript | Page 17 |
| 7 | It is better to give **more detail about baseline NIHSS, mRS, BI, co-morbidities, and co-medication** | Mainly the assessment was conducted to assess spasticity level (MAS), voluntary control of the upper limb (VCG), and hand functions (FMA-UL and ARAT). Baseline NIHSS, mRS, BI were not taken into the account. They were assessed when the patient was admitted to the hospital as an acute patient.  The details about co-morbidities are included under the ‘Results’ section.  The details on the medications that are used for co-morbidities are also included in the data sheet.  A statement is included in the result section stating that ‘These included patients were taking medications only for their present co-morbidities. They were not under any medications to treat spasticity at the stage of patient recruitment and they didn’t take any anti-spastic drugs throughout the treatment protocol. They were only treated with either rESWT or TENS for spasticity management’. | Page 9 |

It will be a great pleasure if you kindly go through these responses for reviewers’ comments and consider this manuscript for publication in your journal.

Thank you.

Yours sincerely,

Mrs. M.K.I.D. Senarath

Mr. S.T.R.D. Thalwaththe

Prof. L.P.M.M.K. Pathirage

Prof. S.A.M. Kularatne

* Corresponding author: M. K. I. D. Senarath, Department of physiotherapy, Faculty of Allied Health Sciences, University of Peradeniya, Peradeniya, 20400, Sri Lanka; Tel: +94772379970; Email: [dilharisenarath@ahs.pdn.ac.lk/](mailto:dilharisenarath@ahs.pdn.ac.lk/) dilsenarath7@gmail.com
