# Supplementary material for: The effectiveness of radial extracorporeal shock wave therapy vs transcutaneous electrical nerve stimulation in the management of upper limb spasticity in chronic-post stroke hemiplegia–A randomized controlled trial
Source: PLoS One. 2023 May 26;18(5):e0283321. doi: 10.1371/journal.pone.0283321 (PMC10218748; doi:10.1371/journal.pone.0283321)
Supplement: Attachment — Submitted filename: R2-Response to reviewers.docx [file pone.0283321.s006.docx]

MKI Dilhari Senarath

Dept. of Physiotherapy

Faculty of Allied Health Sciences

University of Peradeniya

Peradeniya, 20400, Sri Lanka

14/07/2022

Dr. Emily Chenette

Editor-in-Chief

The Journal of PLOS ONE

Dear Dr. Chenette,

**RE:** **Revision 2**-**Response to reviewers’ comments for the article titled ‘The effectiveness of radial Extracorporeal Shock Wave Therapy vs Transcutaneous Electrical Nerve Stimulation in the management of Upper Limb spasticity in chronic-post stroke hemiplegia – A randomized controlled trial’**

Here I am attaching the table regarding the authors’ responses to reviewers’ comments one by one on the above-mentioned article.

|  | Comments to the author | Reviewers’ response | Authors response |
| --- | --- | --- | --- |
| 1 | If the authors have adequately addressed your comments raised in a previous round of review and you feel that this manuscript is now acceptable for publication, you may indicate that here to bypass the “Comments to the Author” section, enter your conflict of interest statement in the “Confidential to Editor” section, and submit your "Accept" recommendation. | Reviewer #1: (No Response) | Not applicable |
| 2 | Is the manuscript technically sound, and do the data support the conclusions?  The manuscript must describe a technically sound piece of scientific research with data that supports the conclusions. Experiments must have been conducted rigorously, with appropriate controls, replication, and sample sizes. The conclusions must be drawn appropriately based on the data presented. | Reviewer #1: No | Thank you for the comment. We have revised our conclusions based on the results that we have obtained.  We included effect sizes to interpret the results in the abstract and the results section. |
| 3 | Has the statistical analysis been performed appropriately and rigorously? | Reviewer #1: (No Response) | Not applicable |
| 4 | Have the authors made all data underlying the findings in their manuscript fully available?  The [PLOS Data policy](http://www.plosone.org/static/policies.action#sharing) requires authors to make all data underlying the findings described in their manuscript fully available without restriction, with rare exception (please refer to the Data Availability Statement in the manuscript PDF file). The data should be provided as part of the manuscript or its supporting information, or deposited to a public repository. For example, in addition to summary statistics, the data points behind means, medians and variance measures should be available. If there are restrictions on publicly sharing data—e.g. participant privacy or use of data from a third party—those must be specified. | Reviewer #1: Yes | Thank you for the response |
| 5 | Is the manuscript presented in an intelligible fashion and written in standard English?  PLOS ONE does not copyedit accepted manuscripts, so the language in submitted articles must be clear, correct, and unambiguous. Any typographical or grammatical errors should be corrected at revision, so please note any specific errors here. | Reviewer #1: Yes | Thank you for the response |
| 6 | Review Comments to the Author  Please use the space provided to explain your answers to the questions above. You may also include additional comments for the author, including concerns about dual publication, research ethics, or publication ethics. (Please upload your review as an attachment if it exceeds 20,000 characters) | Reviewer #1: This study aims to compare the effectiveness of rESW (radial Extracorporeal Shock Wave Therapy) and TENS (Transcutaneous Electrical Nerve Stimulation) in patients with upper limb spasticity in chronic-post stroke hemiplegia. The study used a prospective single-blinded randomized controlled trial to compare the effectiveness of the two treatments in terms of spasticity level on the modified Ashworth scale (MAS), voluntary control of the upper limb (VCG), and hand functions (FMA-UL and ARAT). In this study, the patients were recruited using registry data. The treatments were allocated using a block-randomization and patients from different stroke groups were randomized separately. The null hypothesis and alternative hypothesis were clearly spelled out. Overall, the study design is sound and reasonable. The patient characteristics at baseline were summarized and clearly presented.  However, I have several concerns: | Thank you for the response |
|  |  | Major concerns: 1. The main conclusion from this study “The rESWT group has shown immediate after-effects at the end of the modality’s first application in spasticity reduction and improving voluntary control of the upper limb but not by the TENS” was not supported by the data. The statistical analyses of the mean differences clearly showed significant differences between T1 & T0 and T2& T0 in both treatment groups. 2. The analyses of median differences showed no significant differences for TENS group in either T1 & T0 or T2& T0 groups. However, as the MAS is an ordinal variable with a 6-point scale, the median analysis is not appropriate and conclusive here. Mean differences are much more informative than median differences here, therefore the conclusion should be based on the analysis of mean differences. 3. This is a comparative study, direct comparisons of the change in MAS (or VCG) between the two treatment groups at the T1 and T2 stage will be more informative. | 2. As the reviewers suggested, we conducted the analysis of MAS and VCG with mean values, mean difference%, and paired sample t-test.  The results obtained were incorporated in the abstract, results, and discussion sections of the manuscript.  1. According to the above analysis, the statement should be changed as ‘Both groups have shown immediate after-effects at the end of the modality’s first application in spasticity reduction and improving voluntary control of the upper limb but the rESWT group has shown larger improvement compared with TENS.  3. As suggested, direct comparisons of MAS and VCG between rESWT and TENS were conducted. It is incorporated in the abstract and results section. |
|  |  | Minor concerns: 1. The power justification is confusing. Please clarify whether the power calculation is based on the t-test or Wilcoxon-Mann-Whitney test | In G*power,  We used t-tests as the Test family and  Wilcoxon-Mann-Whitney test as the Statistical test for sample size calculation. It was adopted from previous research conducted on rESWT on spasticity reduction.  Page 5 |
| 7 | PLOS authors have the option to publish the peer review history of their article ([what does this mean?](https://journals.plos.org/plosone/s/editorial-and-peer-review-process#loc-peer-review-history)). If published, this will include your full peer review and any attached files.   If you choose “no”, your identity will remain anonymous but your review may still be made public.   **Do you want your identity to be public for this peer review?** For information about this choice, including consent withdrawal, please see our [Privacy Policy](https://www.plos.org/privacy-policy). | Reviewer #1: No | Not applicable for authors’ responses |

It will be a great pleasure if you kindly go through these responses for reviewers’ comments and consider this manuscript for publication in your journal.

Thank you.

Yours sincerely,

Mrs. M.K.I.D. Senarath

Mr. S.T.R.D. Thalwaththe

Prof. L.P.M.M.K. Pathirage

Prof. S.A.M. Kularatne

* Corresponding author: M. K. I. D. Senarath, Department of physiotherapy, Faculty of Allied Health Sciences, University of Peradeniya, Peradeniya, 20400, Sri Lanka; Tel: +94772379970; Email: [dilharisenarath@ahs.pdn.ac.lk/](mailto:dilharisenarath@ahs.pdn.ac.lk/) dilsenarath7@gmail.com
