# Supplementary material for: The effectiveness of radial extracorporeal shock wave therapy vs transcutaneous electrical nerve stimulation in the management of upper limb spasticity in chronic-post stroke hemiplegia–A randomized controlled trial
Source: PLoS One. 2023 May 26;18(5):e0283321. doi: 10.1371/journal.pone.0283321 (PMC10218748; doi:10.1371/journal.pone.0283321)
Supplement: Attachment — Submitted filename: R3-Response to reviewers and editors.docx [file pone.0283321.s007.docx]

MKI Dilhari Senarath

Dept. of Physiotherapy

Faculty of Allied Health Sciences

University of Peradeniya

Peradeniya, 20400, Sri Lanka

05/09/2022

Dr. Emily Chenette

Editor-in-Chief

The Journal of PLOS ONE

Dear Dr. Chenette,

**RE:** **Revision 3**-**Response to reviewers’ comments for the article titled ‘The effectiveness of radial Extracorporeal Shock Wave Therapy vs Transcutaneous Electrical Nerve Stimulation in the management of Upper Limb spasticity in chronic-post stroke hemiplegia – A randomized controlled trial’**

Here I am attaching the table regarding the authors’ responses to reviewers’ comments one by one on the above-mentioned article.

|  | Comments to the author | Authors response |
| --- | --- | --- |
| 1 | Thanks for the authors for the appropriate changes. The authors have made several changes. | Thank you the editors and the reviewers for the kind response. |
| 2 | Some major concern about the number of co-morbidities. | Page 9  Re-arranged the paragraph regarding medical co-morbidities.  Patients 64/106 (60.4%) were affected by other medical co-morbidities, and some may have reported having a single disease and some may have multiple diseases.  The patients who is having co-morbidities have taken as 100% and further explained on their disease diagnosis. 53/64 (82.81%) HTN, 40/64 (62.5%) DM, 12/64 (18.75%) IHD, 3/64 (4.68%) CKD, 1/64 (1.56%) AF, 1/64 (1.56%) DLP were reported as a whole.  Among those, 16/64 (25%) had only HTN, 7/64 (10.93%), had only DM, 11/64 (17.18%) had HTN and DM, 10/64 (15.62%) had HTN, DM, DLP, and 5/64 (7.81%) had HTN, DM, IHD. |
| 3 | The total number of subjects is not match with the description of every categories. | Re-checked the total number of patients written in each category. The total number for each category (variable) is 106.  Treatment option (106)   - rESWT - 53 - TENS – 53   Experimental (rESWT) and control (TENS) groups were further divided according to the stroke type, gender, and affected side.  Stroke type (106)   - rESWT ischemic stroke – 37 - rESWT hemorrhagic stroke – 16 - TENS ischemic stroke – 37 - TENS hemorrhagic stroke – 16   Gender   - rESWT male – 28 - rESWT female – 25 - TENS male – 19 - TENS female - 34   Affected side   - rESWT right side – 35 - rESWT left side – 18 - TENS right side – 33 - TENS left side - 20 |
| 4 | The authors have made changes in the tables, but it is still too complicated. | More columns were added to the tables explaining the results and it is revised to display in a simple manner. |
| 5 | Please revise the limitations of your study. | Thank you for the kind comment.  The limitations of the study paragraph are revised accordingly. |

It will be a great pleasure if you kindly go through these responses for reviewers’ and editors’ comments and consider this manuscript for publication in your journal.

Thank you.

Yours sincerely,

Mrs. I.D. Senarath

Mr. S.T.R.D. Thalwaththe

Prof. L.P.M.M.K. Pathirage

Prof. S.A.M. Kularatne

* Corresponding author: I. D. Senarath, Department of physiotherapy, Faculty of Allied Health Sciences, University of Peradeniya, Peradeniya, 20400, Sri Lanka; Tel: +94772379970; Email: [dilharisenarath@ahs.pdn.ac.lk/](mailto:dilharisenarath@ahs.pdn.ac.lk/) dilsenarath7@gmail.com
