# Supplementary material for: The effectiveness of radial extracorporeal shock wave therapy vs transcutaneous electrical nerve stimulation in the management of upper limb spasticity in chronic-post stroke hemiplegia–A randomized controlled trial
Source: PLoS One. 2023 May 26;18(5):e0283321. doi: 10.1371/journal.pone.0283321 (PMC10218748; doi:10.1371/journal.pone.0283321)
Supplement: Attachment — Submitted filename: R4-Response to reviewers and editors.docx [file pone.0283321.s008.docx]

MKI Dilhari Senarath

Dept. of Physiotherapy

Faculty of Allied Health Sciences

University of Peradeniya

Peradeniya, 20400, Sri Lanka

22/09/2022

Dr. Emily Chenette

Editor-in-Chief

The Journal of PLOS ONE

Dear Dr. Chenette,

**RE:** **Revision 4**-**Response to reviewers’ comments for the article titled ‘The effectiveness of radial Extracorporeal Shock Wave Therapy vs Transcutaneous Electrical Nerve Stimulation in the management of Upper Limb spasticity in chronic-post stroke hemiplegia – A randomized controlled trial’**

Here I am attaching the table regarding the authors’ responses to reviewers’ comments one by one on the above-mentioned article.

|  | Comments to the author | Authors response |
| --- | --- | --- |
| 1 | Thank you for the responses. I still concern about the data in table 3 and table 4. I ask the authors to review the data again. Some mean differences in table 3 and 4 is much higher than the final (T2) visit score. It is not possible to have higher mean differences than the final scores. | Thank you the editors and the reviewers for the kind response.  As suggested, we reviewed the data again and did the statistical analysis again.  T0-Baseline values  T1-values just after 1^st^ treatment application  T2-values at the end of the four-week treatment protocol  T0 T1 T2    T0&T1 T1&T2  T0&T2  T1 values are higher than the T0 values as the participants benefited after 1^st^ application of the modality.  But they achieved more outcome values after applying the same modality for four weeks, which we assessed at the end of the four-week protocol, T2.  Therefore, T2 values are higher than both T0 and T1 values.  It is like; T2>T1>T0  If we compare the results with a single application (T1) to the baseline and four applications (T2) to the baseline;  After four applications of the modality patients were presented with great results.  Therefore, if we compare the time pairs, the mean differences between the time pairs are like;  T0&T2>T1&T2>T0&T1  The table 3 and 4 are reporting the results accordingly.  Yes, it is true, the mean difference of any time pair cannot be higher than the score at T2.  In the columns of T0, T1, and T2, we indicate the **mean values** of the subgroups, and in the columns of the time pairs (T0&T1, T0&T2, T1&T2), we presented the **percentage of the mean difference**.  Therefore, values in some of the cells of time pairs can be higher than the T2 value, as they are showing a percentage of the mean difference. |
| 2 | The limitations of your study should be from the methodological point of view (the numbers of samples, the site of the study, the blinding or objective measurement, the intent to treat analysis, the adjustment of important prognostic factors). | Thank you for the comment. We have revised the limitations section accordingly. |

It will be a great pleasure if you kindly go through these responses for reviewers’ and editors’ comments and consider this manuscript for publication in your journal.

Thank you.

Yours sincerely,

Mrs. I.D. Senarath

Mr. S.T.R.D. Thalwaththe

Prof. L.P.M.M.K. Pathirage

Prof. S.A.M. Kularatne

* Corresponding author: I. D. Senarath, Department of physiotherapy, Faculty of Allied Health Sciences, University of Peradeniya, Peradeniya, 20400, Sri Lanka; Tel: +94772379970; Email: [dilharisenarath@ahs.pdn.ac.lk/](mailto:dilharisenarath@ahs.pdn.ac.lk/) dilsenarath7@gmail.com
