# Supplementary material for: The effectiveness of radial extracorporeal shock wave therapy vs transcutaneous electrical nerve stimulation in the management of upper limb spasticity in chronic-post stroke hemiplegia–A randomized controlled trial
Source: PLoS One. 2023 May 26;18(5):e0283321. doi: 10.1371/journal.pone.0283321 (PMC10218748; doi:10.1371/journal.pone.0283321)
Supplement: Attachment — Submitted filename: R5-Response to reviewers and editors.docx [file pone.0283321.s009.docx]

MKI Dilhari Senarath

Dept. of Physiotherapy

Faculty of Allied Health Sciences

University of Peradeniya

Peradeniya, 20400, Sri Lanka

12/10/2022

Dr. Emily Chenette

Editor-in-Chief

The Journal of PLOS ONE

Dear Dr. Chenette,

**RE:** **Revision 5**-**Response to reviewers’ comments for the article titled ‘The effectiveness of radial Extracorporeal Shock Wave Therapy vs Transcutaneous Electrical Nerve Stimulation in the management of Upper Limb spasticity in chronic-post stroke hemiplegia – A randomized controlled trial’**

Here I am attaching the table regarding the authors’ responses to reviewers’ comments one by one on the above-mentioned article.

|  | Comments to the author | Authors response |
| --- | --- | --- |
| 1 | Please mention the aim of your study. It is uncommon to state the hypothesis and null hypothesis. | Thank you for the comment. We remove the hypothesis from the manuscript.  The aim of the study is already mentioned in the last sentence of the second paragraph of the introduction section.  Page 3, Line 69-71 |
| 2 | Some mean difference are more than 50% and 100%. For example the T0 is 6, and the T2 is 32.  Can you tell me the mean difference in table? | Thank you for the comment.  The scores of the MAS and VCG assessment tools range from 0-6. Therefore the mean scores of spasticity grading and voluntary control grading at T0, T1, and T2 time points lie between 0-6 only.  But, in the FMA-UL tool, the score range from 0 to a total of 66, and in the ARAT scale, the score range from 0 to 57.  Therefore the mean scores of FMA-UL and ARAT at T0, T1, and T2 can lie between 0-66 and 0-57 respectively.  The **mean scores** of MAS and VCG at T0, T1, and T2 are mentioned in the first three columns of Tables 2 and 3 respectively.  The **mean scores** of FMA-UL and ARAT at T0, T1, and T2 are mentioned in the first three columns of Tables 4 and 5 respectively.  To evaluate the improvement by the intervention, we took the **percentage of mean difference at each time point**; T0 & T1, T0 & T2, and T1 & T2.  Even though the tools have different scales, as we are taking the percentage of mean difference, it will allow the readers to easily compare the results among sub-groups. |
| 3 | There should be abbreviations in every table. | We add abbreviations to all the tables accordingly. |

It will be a great pleasure if you kindly go through these responses for reviewers’ and editors’ comments and consider this manuscript for publication in your journal.

Thank you.

Yours sincerely,

Mrs. I.D. Senarath

Mr. S.T.R.D. Thalwaththe

Prof. L.P.M.M.K. Pathirage

Prof. S.A.M. Kularatne

* Corresponding author: I. D. Senarath, Department of physiotherapy, Faculty of Allied Health Sciences, University of Peradeniya, Peradeniya, 20400, Sri Lanka; Tel: +94772379970; Email: [dilharisenarath@ahs.pdn.ac.lk/](mailto:dilharisenarath@ahs.pdn.ac.lk/) dilsenarath7@gmail.com
